# Aberrant DNA methylation of *PTPRG* as one possible mechanism of its under‐expression in CML patients in the State of Qatar

**DOI:** 10.1002/mgg3.1319

**Published:** 2020-07-23

**Authors:** Mohamed A. Ismail, Muthanna Samara, Ali Al Sayab, Mohamed Alsharshani, Mohamed A. Yassin, Govindarajulu Varadharaj, Marzia Vezzalini, Luisa Tomasello, Maria Monne, Hisham Morsi, M. Walid Qoronfleh, Hatem Zayed, Richard Cook, Claudio Sorio, Helmout Modjtahedi, Nader I. Al‐Dewik

**Affiliations:** ^1^ School of Life Science, Pharmacy and Chemistry Faculty of Science, Engineering & ComputingFaculty of Science, Engineering & Computing Kingston University London Kingston‐Upon‐Thames UK; ^2^ Interim Translational Research Institute (iTRI) Hamad Medical Corporation (HMC) Doha Qatar; ^3^ Department of Psychology Kingston University London Kingston upon Thames, London UK; ^4^ Diagnostic Genetics Division (DGD) Department of Laboratory Medicine and Pathology (DLMP) Hamad Medical Corporation (HMC) Doha Qatar; ^5^ Department of Medical Oncology National Centre for Cancer Care and Research Hamad Medical Corporation (HMC) Doha Qatar; ^6^ Genetrics Inc Dubai United Arab Emirates; ^7^ General Pathology Division Department of Medicine University of Verona Verona Italy; ^8^ Wexner Medical Center Biomedical Research Tower The Ohio State University Columbus OH USA; ^9^ Centro di Diagnostica Biomolecolare e Citogenetica Emato‐Oncologica San Francesco” Hospital Nuoro Italy; ^10^ Quality of Life unit National Center for Cancer Care and Research, (NCCCR) Hamad Medical Corporation (HMC) Doha Qatar; ^11^ World Innovation Summit for Healthcare (WISH) Qatar Foundation Doha Qatar; ^12^ Department of Biomedical Sciences Biomedical Research Center College of Health Sciences QU Health Qatar University Doha Qatar; ^13^ Qatar Medical Genetic Center (QMGC) Hamad General Hospital (HGH) and Interim Translational Research Institute (iTRI) Hamad Medical Corporation (HMC) Doha Qatar; ^14^ College of Health and Life Science (CHLS) Hamad Bin Khalifa University (HBKU) Doha Qatar; ^15^ Department of Pediatrics Women's Wellness and Research Center (WWRC) HMC Doha Qatar

**Keywords:** aberrant DNA, cancer, CML, epigenetics, methylation, PTPRG, Qatar

## Abstract

**Background:**

Several studies showed that aberrant DNA methylation is involved in leukemia and cancer pathogenesis. Protein tyrosine phosphatase receptor gamma (PTPRG) expression is a natural inhibitory mechanism that is downregulated in chronic myeloid leukemia (CML) disease. The mechanism behind its downregulation has not been fully elucidated yet.

**Aim:**

This study aimed to investigate the CpG methylation status at the *PTPRG* locus in CML patients.

**Methods:**

Peripheral blood samples from CML patients at time of diagnosis [no tyrosine kinase inhibitors (TKIs)] (*n* = 13), failure to (TKIs) treatment (*n* = 13) and healthy controls (*n* = 6) were collected. DNA was extracted and treated with bisulfite treatment, followed by PCR, sequencing of 25 CpG sites in the promoter region and 26 CpG sites in intron‐1 region of *PTPRG*. The bisulfite sequencing technique was employed as a high‐resolution method.

**Results:**

CML groups (new diagnosed and failed treatment) showed significantly higher methylation levels in the promoter and intron‐1 regions of *PTPRG* compared to the healthy group. There were also significant differences in methylation levels of CpG sites in the promoter and intron‐1 regions amongst the groups.

**Conclusion:**

Aberrant methylation of *PTPRG* is potentially one of the possible mechanisms of *PTPRG* downregulation detected in CML.

## Graphical abstract

This study delivers important message that the epigenetic silencing phenomenon via DNA hyper‐methylation resulting in decreased PTPRG protein expression which play a critical role in CML disease initiation and progression. These findings also highlight the importance of evaluation and characterization the methylation levels of PTPRG gene in CML patients worldwide. Increasing cancer societies’ awareness on PTPRG methylation and its silencing effects in CML can be enriched and integrated into the international CML patients’ management.

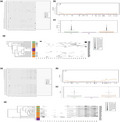

## 1 BACKGROUND

Chronic myeloid leukemia (CML) is a clonal myeloid stem cell disorder associated with abnormal exponential proliferation of granulocytes and their precursors (Goldman, 2010). CML incidence rates are estimated to be 0.7–1.0/100,000 (Hoglund, Sandin, & Simonsson, 2015). *BCR‐ABL1* fusion oncogene that results from the Philadelphia (Ph^+^) chromosome *t* (9; 22) (q34; q11), is the hallmark of CML disease. The translocation juxtaposes the *c‐abl* (ABL1) gene on chromosome 9 with the breakpoint cluster region (*BCR*) gene on chromosome 22. The BCR‐ABL oncoprotein plays a key role of the constitutive activation of the tyrosine kinase domain (Aladag & Haznedaroglu, 2019; Deininger, Goldman, & Melo, 2000), therefore it has become an important target for therapeutic interventions with small molecule tyrosine kinase inhibitors (TKIs) which compete with the ATP binding site of the catalytic domain of ABL tyrosine kinase (Soverini, Bassan, & Lion, 2019). In the past decade, the introduction of these TKIs has demonstrated a significant improvement in managing and treating CML. At present five TKIs; Imatinib Mesylate (IM), Nilotinib, Dasatinib, Ponatinib, and Bosutinib are approved by the United States Food and Drug Administration (FDA) for the treatment of CML patients (Druker et al., 2006; Gover‐Proaktor et al., 2019; Jabbour & Kantarjian, 2016; Sasaki et al., 2016). Despite the safety and efficacy of these TKIs in achieving major / complete response at the molecular, cytogenetic, and hematological levels (Baccarani, Rosti, & Soverini, 2019; Druker et al., 2006; Hochhaus et al., 2007), a substantial proportion of patients (around 20%–25%) develop resistance to treatments (Al‐Dewik, Jewell, Yassin, & Morsi, 2015; Apperley, 2007; Baccarani et al., 2019). TKIs resistance that is *BCR‐ABL1*‐dependent, where mechanisms such as point mutations or cellular/biological processes that interfere with TKI bioavailability disrupt the effectiveness of BCR‐ABL1 kinase inhibition. Moreover, BCR‐ABL1‐independent resistance is attributed to alternative signaling pathways operating in the presence of effective TKI inhibition of *BCR‐ABL1* (Al‐Dewik, Ayoubi et al., 2011; Kalle, Sachchidanand, & Pallu, 2010; Nambu et al., 2010; Patel, O'Hare, & Deininger, 2017). One of the potential independent inhibitory mechanisms is the epigenetic regulation of protein tyrosine phosphatases.

Epigenetic silencing is a phenomenon whereby gene transcription is suppressed through DNA methylation (a process that may regulate gene function) resulting in decreased protein expression. Several studies have suggested that hypermethylation might play a role in disease progression in CML. Hypermethylation of several genes was associated with the progression of CML, its pathogenesis and response to treatment (Heller et al., 2016; Jelinek et al., 2011; Machova Polakova, 2013; Toyota et al., 2001; Wang et al., 2009, 2010).

Protein tyrosine phosphatase (PTP) superfamily of enzymes represents a natural regulatory mechanism of the tyrosine kinase family. They have the ability to remove phosphate groups from phosphorylated tyrosine residues leading to an equilibrium status in normal populations. Based on their cellular localization, PTPs are classified as receptor‐like and nonreceptor. Even though receptor‐type protein tyrosine phosphatases (PTPRs) share similar basic structure, distinct PTPRs have specific targets and, may thus, play alternative roles in cell regulation (Du & Grandis, 2015; Jiang, den Hertog, & Hunter, 2000; Tonks, 2006). Of these, protein tyrosine phosphatase receptor gamma (*PTPRG*) was described as a tumor suppressor gene in several tumors and its expression level was found to be significantly downregulated in CML patients and correlates with its promoter methylation in both patients and cell lines (Della Peruta, 2010; Tomasello et al., 2020). Moreover, PTPRG expression is restored in patients who respond optimally to TKIs treatment, and its expression remains low in patients who fail treatment (Vezzalini et al., 2017). More recently, we identified a single Nucleotide Polymorphism (SNPs) (rs62620047) in *PTPRG* (Y92H) in patients who failed Imatinib Mesylate (IM) treatment (Al‐Dewik et al., 2016). While the molecular and flow cytometry characteristics of *PTPRG* were studied (Vezzalini et al., 2017), the contributing epigenetic mechanisms that influence the *PTPRG* activity in CML patients remains unclear and warrants further investigation.

The aim of this study is to investigate the methylation patterns of *PTPRG* gene in a cohort of CML patients in Qatar where resistance to IM treatment has been reported to be significantly higher than other parts of the world (Al‐Dewik, Jewell, Yassin, El‐Ayoubi, & Morsi, 2014).

## 2 MATERIALS AND METHODS

### 2.1 Patient recruitment, characteristics, and sample collection

Informed consent was obtained from all participants. The study was approved by both Ministry of Public Health and institutional review board of Hamad Medical Corporation (Project No. 11118/11). This study adhered to the World Medical Association's Declaration of Helsinki (1964–2008) and its amendments for Ethical Human Research including confidentiality, privacy, and data management. A total of 26 adult CML patients and 6 matched healthy controls that were confirmed to have normal complete blood count (CBC) and negative for *BCR‐ABL1* translocation were included in this study.

The peripheral blood samples were collected in EDTA tubes for newly diagnosed patients before starting TKIs and at time of failure for patients who relapsed or failed treatment according to European Leukemia Net (ELN) guidelines 2013. The CML patients' response to TKIs treatment was assessed based on the hematologic, cytogenetic, and molecular response results according to (ELN 2013) (Baccarani et al., 2013; Steegmann et al., 2016). Resistance to treatment was defined as showing lack of one of the following; hematological response and/or Ph^+^ >95% by 3 months, *BCR‐ABL1* >10% and/or Ph^+^ >35% by 6 months, *BCR‐ABL1* >1% and/or Ph^+^ >0 by 12 months after the start of treatment.

### 2.2 DNA isolation and bisulfite conversion

Total DNA was isolated from total leucocytes with Maxwell^®^ 16 DNA Purification Kits as per manufacture guidelines (Khokhar, Mitui, Leos, Rogers, & Park, 2011). Purity of extracted DNA was assessed by a Nano Drop spectrophotometer 2000 (Thermofisher Scientific), a ratio of 1.8–2.4 was considered acceptable and for optimal results, an absolute quantity of 200–500 ng of DNA was used.

The DNA samples were then treated with sodium bisulfite according to the manufacturer's instructions (EpiTect Bisulfite Kit, QIAGEN). The bisulfite treatment catalyzes the deamination of all the unmethylated cytosine (uC), nucleotides to uracil (U), or thymidine (T) nucleotides and leaves the methylated cytosine (mC) unchanged. For optimal results, the amount of starting DNA in the bisulfite modification process was kept at 200–500 ng.

### 2.3 Primers design, bisulfite sequencing PCR (BSP), and Gradient Polymerase Chain reaction

The University of California, Santa Cruz (UCSC) Genome Browser (UCSC, 2013) was utilized to identify the possible “CpG sites” flanking the cytosine phosphate guanine (CpG) region followed by “Bio Edit Sequence Aliqment Editor” tool to identify the forward and reverse primers. Finally, BiSearch is a primer‐design and search tool utilized to ensure amplification of specific PCR products (Table S1) (BiSearch, 2019). Gradient PCR was performed to find the specific annealing temperature for the selected gene. Specific products 321bp and 218bp were detected at 60°C for promoter and intron‐1 regions of *PTPRG,* respectively. Bisulfite treatment was performed followed by Sanger sequencing (Table S2).

### 2.4 Bisulfite sequencing

The PCR products were sequenced with an ABI PRISM BigDye terminator sequencing kit v1.1 (Life Technologies) and directly analyzed by an automated ABI 3130 Genetic Analyzer (Life Technologies).

### 2.5 Methylation analysis

The analysis of the methylation status of CpG sites in the region amplified by PCR was performed using the ESME (Epigenetic Sequencing Methylation) Analysis Software (Lewin, Schmitt, Adorjan, Hildmann, & Piepenbrock, 2004). The software uses a genomic sequence as a reference and four‐dye trace sequencing data as tests. The ratio of C to T at CG sites was determined after correction for incomplete conversion to determine extent of methylation. Furthermore, the percentage of methylation was calculated as the peak height of C versus the peak height of C plus the peak height of T for each CpG site as shown in the computer‐generated sequencing chromatogram extracted from the Chromas program (Version 2.32, Technelysium). The ABI sequencing data files (*.ab1) along with reference fasta‐files (*.fa) were run through the ESME software. A single C at the corresponding CpG site was considered as 100% methylation, a single T as no methylation and overlapping C and T as partial methylation (0%–100%) (Jiang et al., 2010).

Cytosine phosphate guanine sites of promoter and intron‐1 regions of *PTPRG* were plotted using Methylation plotter (Mallona, Díez‐Villanueva, & Peinado, 2014). The methylation levels (0%–100%) were converted to (0–1) for plotting purposes (Mallona, Diez‐Villanueva, & Peinado, 2014). The genomic co‐ordinates were identified for the CpG sites (Table S3). The methylation data obtained was represented diagrammatically as follows: a horizontal series represented a single sample, circles referred to CpG sites and shape filled indicated the methylation of the site.

### 2.6 *BCR‐ABL1* and *PTPRG* quantification by RQ‐PCR

The *BCR‐ABL1* and *PTPRG* quantification were carried out using RQ‐PCR as previously described (Al‐Dewik et al., 2014; Della Peruta et al., 2010; Piras et al., 2013; Vezzalini et al., 2017) (Table 1).

**Table 1:** CML patients’ characteristics according to gender, age, clinical phase, the type and total dose of TKIs received and response to treatments

Patients	Gender Male (M), Female (F)	Age (years)	Clinical phase	BCR‐ABL1(IS)	PTPRG/ABL*100		Treatment	Response
CML case 01.	M	45	CP	100%	0.02%	No treatment.		Newly diagnosed
CML case 02.	M	23	CP	100%	0.01%	No treatment.		Newly diagnosed
CML case 03.	M	28	CP	100%	0.01%	No treatment.		Newly diagnosed
CML case 04.	M	38	CP	100%	0.01%	No treatment.		Newly diagnosed
CML case 05.	M	43	AP	100%	0.01%	No treatment.		Newly diagnosed
CML case 06.	F	45	CP	100%	0.01%	No treatment.		Newly diagnosed
CML case 07.	M	46	CP	100%	0.02%	No treatment.		Newly diagnosed
CML case 08.	F	28	CP	100%	0.01%	No treatment.		Newly diagnosed
CML case 09.	M	40	CP	100%	0.01%	No treatment.		Newly diagnosed
CML case 10.	M	34	CP	100%	0.01%	No treatment.		Newly diagnosed
CML case 11.	M	58	CP	100%	0.01%	No treatment.		Newly diagnosed
CML case 12.	F	43	CP	100%	0.01%	No treatment.		Newly diagnosed
CML case 13.	M	32	CP	100%	0.01%	No treatment.		Newly diagnosed
CML case 14.	F	49	CP	37%	0.2%	Imatinib (400 mg) No changes in treatment		Failed treatment
CML case 15.	F	35	CP	86%	0.01%	Imatinib(400 mg), then shift to Dasatinib (50 mg)		Failed treatment
CML case 16.	M	23	CP	35%	0.3%	Imatinib (400 mg) No changes in treatment		Failed treatment
CML case 17.	M	25	CP	12%	0.3%	Imatinib (400 mg) No changes in treatment		Failed treatment
CML case 18.	F	34	CP	45%	0.2%	Imatinib (400 mg) No changes in treatment		Failed treatment
CML case 19.	M	31	CP	33%	0.2%	Imatinib (400 mg) No changes in treatment		Failed treatment
CML case 20.	F	29	CP	25%	0.3%	Imatinib (400 mg) No changes in treatment		Failed treatment
CML case 21.	F	35	CP	68%	0.1%	Imatinib (400 mg) No changes in treatment		Failed treatment
CML case 22.	M	38	CP	80%	0.02%	Imatinib (400 mg) No changes in treatment		Failed treatment
CML case 23.	M	38	CP	60%	0.1%	Imatinib (400 mg) No changes in treatment		Failed treatment
CML case 24.	M	34	CP	55%	0.1%	Imatinib (400 mg)		Failed treatment
CML case 25.	M	61	CP	11%	0.4%	Nilotinib (300 mg) No changes in treatment		Failed treatment
CML case 26.	M	40	BC	15%	0.3%	Dasatinib (70 mg) No changes in treatment		Failed treatment

Abbreviations: AP, accelerated phase; BC; blast crisis phase; CP, chronic phase.

### 2.7 Statistical analysis

Descriptive statistics in the form of median range and frequency, and percentages were calculated. For continuous outcomes and categorical independent variables, *T* test for independent samples was used to test the mean differences for two groups and one‐way ANOVA with Bonferroni post hoc analysis was employed to test the mean differences for three groups using SPSS 24. All *p* values presented are two‐tailed, and *p* values < .05 are considered statistically significant.

## 3 RESULTS

### 3.1 Participants’ characteristics

Out of the 26 CML patients, 13 were newly diagnosed (ND) with CML and 13 failed treatment (F) (Table 1). Patients’ age ranged from 25–60 years (mean 37.48 and *SD*: 9.82) with a male to female ratio of 18 (69.2%) males and 8 (30.8%) females. In addition, there were six healthy participants who have never had cancer (H) (Age range: 23–46 years mean: 37.17; *SD*: 9.58; Gender: five (83.3%) male and one (16.7%) female). Out of the 26 patients, 24 patients were in Chronic Phase (CP) (92.3%), one patient was in accelerated phase (AP) (3.85%) and one patient was in Blast Crisis (BC) phase (3.85%).

### 3.2 Hypermethylation of the promoter region of *PTPRG*

*T* test was performed to study the methylation pattern of promoter of *PTPRG* in both cases and controls. The results revealed a significant difference in promoter methylation levels between CML (ND and F groups) and the healthy group (*t* (30) = 5.7, *p* < .001) (CML: mean = 6.77, *SD*: 2.87; Healthy: mean = 0.00, *SD*: 0.00).

Additionally, we tested the differences between the three groups (ND, F and H). One‐way ANOVA and Bonferroni post hoc test results indicated that the ND and F groups had significantly higher methylation compared with the H group (*p* < .001). There was no significant difference between ND and F groups (Table 2).

**Table 2:** Descriptive analysis for methylation levels of the Promoter region and Intron‐1 region

Region	Groups	*N*	Mean ± *SD*	95% confidence interval for mean	
				Lower bound	Upper bound
Promotor	Newly Diagnosed (ND)	13	7.3 ± 3.0	5.5	9.2
	Failed (F)	13	6.1 ± 2.6	4.5	7.7
	Healthy (H)	6	0.00	0.00	0.00
Intron‐1	Newly Diagnosed (ND)	13	14.13 ± 3.6	11.95	16.312
	Failed (F)	13	15.11 ± 3.3	13.147	17.08
	Healthy (H)	6	0.00	0.00	0.00

### 3.3 Methylation patterns in the 25 CpG sites of promoter region of *PTPRG*

**Figure 1:**
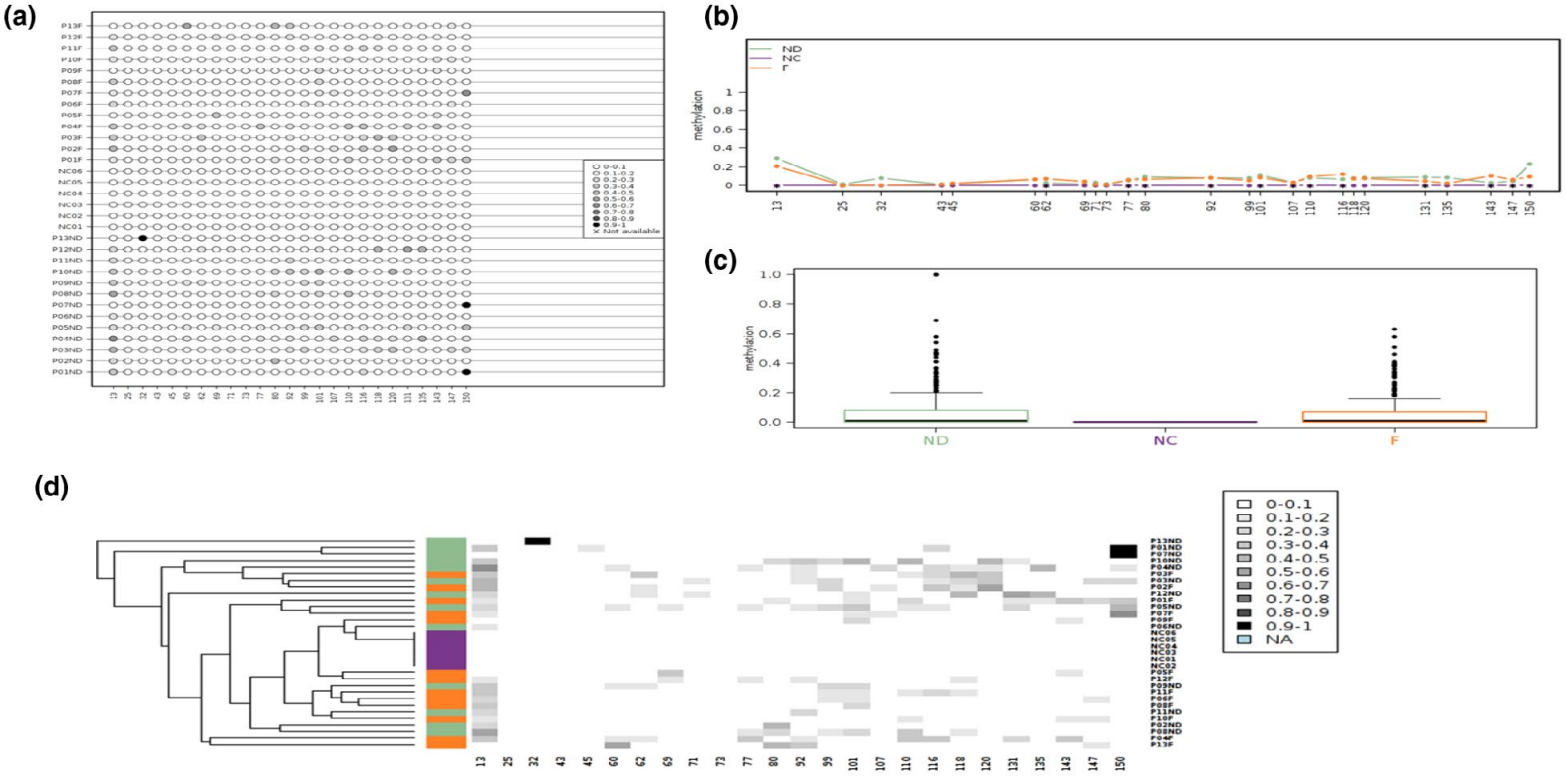
Data visualization with Methylation plotter for 25 sites of Promoter region of *PTPRG*. (a) Lollipop‐like visualization of methylation sites. (b) Methylation profiling plot reflecting with asterisks those positions for which significant differences between groups were detected. (c) Boxplots for each group showing the methylation data distribution. (d) Unsupervised hierarchical clustering of the data; sample label colors reflect groups classification

One‐way ANOVA was performed to compare methylation status in each CpG site in the promoter region between the three groups (ND, F, and H). There were significant differences in 2 out of 25 CpG sites (13 and 143) among the groups (*F* (2, 29) = 7.0; *p* = .003 and *F* (2, 29) = 4.35*; p* = .022 respectively). Bonferroni post hoc test results indicated that methylation in CpG 13 for the ND and the F groups was significantly higher compared to the H group (*p* = .002 and *p* = .035 respectively). Furthermore, methylation in CpG 143 for the F group was significantly higher compared to the H group (*p* = .045). (Figure 1). No significant differences were found in the rest of the CpG sites.

### 3.4 Hypermethylation of intron‐1 region of *PTPRG*

*T* test was performed to study the methylation pattern of intron‐1 of *PTPRG* in both cases and controls. The results revealed a significant difference in intron‐1 methylation levels between CML (ND and F groups) and the healthy group (*t* (30) = 10.38, *p* < .001) (CML: mean = 14.62, *SD*: 3.41; Healthy: mean = 0.00, *SD*: 0.00).

One‐way ANOVA showed significant differences in methylation between the three groups (ND, F and H) for intron‐1 region [*F* (2, 29) = 53.590, *p* = .001]. Bonferroni post hoc test results indicated that the methylation status for the ND and the F groups was significantly higher than the H group (*p* < .001). There was no significant difference between the ND and the F groups (Table 2).

### 3.5 Methylation patterns in 26 CpG sites of Intron‐1 region of *PTPRG*

**Figure 2:**
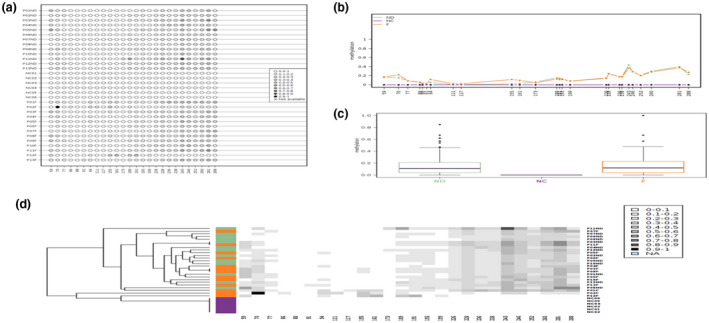
Data visualization with Methylation plotter for 26 sites of Intron‐1 region of *PTPRG*. (a) Lollipop‐like visualization of methylation sites. (b) Methylation profiling plot reflecting with asterisks those positions for which significant differences between groups were detected. (c) Boxplots for each group showing the methylation data distribution. (d) Unsupervised hierarchical clustering of the data; sample label colors reflect the groups classification

One‐way ANOVA was also performed to compare methylation levels in each CpG sites in the intron‐1 region between the three groups; ND, F, and H. The results indicated that there were significant differences in 23 out of 26 CpG sites (Figure 2 and Table 3). Bonferroni post hoc test revealed that the methylation levels were significantly higher amongst the ND and the F groups compared to the H group in most of the intron‐1 CpG sites except CpG 70, CpG 94, CpG 155 and CpG 161 (in the ND group) and CpG 173 (in the F group). In addition, the F group had significantly higher methylation levels in the CpG sites 94 (*p* = .003) and 155 (*p* = .01) compared to the ND group.

**Table 3:** Methylation levels of the 23 CpG sites in the Intron‐1 region amongst F and ND groups compared to H group

Site	Groups	Mean ± *SD*	95% confidence interval range	*p* value
CpG 59	F	17.00 ± 10.47	10.67 ± 23.32	.013
	ND	16.07 ± 13.66	7.82 ± 24.33	.019
CpG 70	F	21.38 ± 24.21	6.75 ± 36.02	.034
	ND	15.00 ± 5.89	11.44 ± 18.56	.204
CpG 77	F	8.30 ± 5.089	5.23 ± 11.38	.001
	ND	8.30 ± 3.79	6.01 ± 10.60	.001
CpG 86	F	5.46 ± 3.69	3.23 ± 7.69	.001
	ND	4.62 ± 1.61	3.64 ± 5.59	.003
CpG 88	F	2.69 ± 2.39	1.25 ± 4.14	.027
	ND	2.85 ± 1.86	1.72 ± 3.97	.018
CpG 91	F	1.92 ± 3.59	−0.25 ± 4.09	.352
	ND	1.00 ± 1.08	0.34 ± 1.65	1.000
CpG 94	F	11.38 ± 6.70	7.33 ± 15.43	.000
	ND	4.92 ± 2.22	3.58 ± 6.26	.109
CpG 111	F	2.08 ± 6.64	−1.94 ± 6.09	1.000
	ND	0.15±0.55	−0.18±0.49	1.000
CpG 117	F	1.84 ± 5.18	−1.29 ± 4.98	.817
	ND	0.23±0.44	−0.03±0.50	1.000
CpG 155	F	11.30 ± 11.78	4.19 ± 18.43	.016
	ND	1.77 ± 1.48	0.87 ± 2.66	1.000
CpG 161	F	9.46 ± 11.23	2.68 ± 16.25	.041
	ND	2.69 ± 1.75	1.63 ± 3.75	1.000
CpG 173	F	4.00 ± 2.97	2.204 ± 5.80	.169
	ND	5.85 ± 5.59	2.46 ± 9.22	.021
CpG 189	F	12.38 ± 6.51	8.45 ± 16.32	.042
	ND	15.07 ± 13.44	6.95 ± 23.20	.011
CpG 191	F	13.85 ± 10.97	7.22 ± 20.47	.002
	ND	10.92 ± 3.33	8.91 ± 12.93	.016
CpG 193	F	11.38 ± 4.37	8.74 ± 14.02	.000
	ND	12.70 ± 4.73	9.83 ± 15.55	.000
CpG 199	F	8.00 ± 4.14	5.50 ± 10.50	.000
	ND	7.85 ± 4.02	5.42 ± 10.27	.001
CpG 226	F	14.62 ± 7.10	10.32 ± 18.90	.000
	ND	13.62 ± 4.23	11.06 ± 16.17	.000
CpG 228	F	23.62 ± 9.82	17.68 ± 29.55	.000
	ND	24.46 ± 6.92	20.28 ± 28.65	.000
CpG 236	F	17.62 ± 9.22	12.05 ± 23.18	.000
	ND	17.62 ± 5.58	14.25 ± 20.98	.000
CpG 238	F	17.54 ± 9.47	11.81 ± 23.26	.000
	ND	17.62 ± 5.55	14.26 ± 20.97	.000
CpG 243	F	36.23 ± 13.23	28.24 ± 44.23	.000
	ND	43.23 ± 14.76	34.21 ± 52.15	.000
CpG 246	F	28.54 ± 10.71	22.07 ± 35.01	.000
	ND	28.54 ± 7.48	24.02 ± 33.06	.000
CpG 252	F	19.77 ± 8.05	14.90 ± 24.64	.000
	ND	20.23 ± 4.21	174.70 ± 22.77	.000
CpG 260	F	28.00 ± 11.23	21.21 ± 34.79	.000
	ND	29.69 ± 7.03	25.45 ± 33.94	.000
CpG 281	F	37.23 ± 15.54	27.84 ± 46.62	.000
	ND	39.69 ± 14.37	31.01 ± 48.38	.000
CpG 288	F	27.31 ± 12.60	19.70 ± 34.92	.000
	ND	22.77 ± 12.50	15.21 ± 30.33	.001

## 4 DISCUSSION

This is the first prospective study to evaluate epigenetic mechanisms of *PTPRG* regulation amongst CML patient's population where the rate of IM resistances is higher than other reported parts of the world (Al‐Dewik, Ayoubi et al., 2011; Al‐Dewik et al., 2014; Al‐Dewik, Jewell et al., 2014; Al‐Dewik, Morsi et al., 2016; Patel et al., 2017). It addresses the important role of *PTPRG* as a regulatory element in *BCR‐ABL1*‐mediated oncogenesis. Our study provides an evidence of the involvement of the epigenetic modification of *PTPRG* in the pathogenesis of CML. *PTPRG* was found to be significantly hyper methylated compared to the control (Figures 1 and 2).

Protein tyrosine phosphatase receptor gamma is known to induce a reduction of protein BCR‐ABL‐specific tyrosine phosphorylation of its direct downstream targets/substrates such as CRKL and of STAT5 (Della Peruta et al., 2010). In the current study, we expanded the methylation coverage of *PTPRG* via studying two regions of its promoter; 321bp andintron‐1 218 bp using advanced molecular technique such as Sanger sequencing. In the same context, Della Peruta *et al.,* documented earlier that upregulated PTPRG expression is associated with a reduction in methylation levels in 166 bp of *PTPRG* using Methylation‐specific PCR technique (Della Peruta et al., 2010). More recently, we demonstrated that in *PTPRG*‐negative CML cell lines, the methylating enzyme DNA (cytosine‐5)‐methyl transferees 1 (DNMT1) is over‐expressed, bind to *PTPRG* promoter and is responsible for its hypermethylation, while its inhibition or downregulation correlates with *PTPRG* re‐expression (Tomasello et al., 2020). Our findings revealed that the methylation occurs more frequently in the intron‐1 region compared to the promoter region in CML patients besides showing a significant increase of the methylated percentage at the CpG sites in both promoter and intron‐1 regions compared to healthy individuals (Table 3). Interestingly, our findings indicated and confirmed that the hyper methylated pattern of *PTPRG* gene in CML patients acts as an early promoter for CML formation and to be dependent on *BCR‐ABL1* titers. It may well contribute as a *BCR‐ABL1* independent resistance molecular event.

We analyzed 51 CpG sites in *PTPRG* in CML and healthy control groups for methylation. Overall, the frequency of methylated CpG sites was significantly higher in CML cases compared to healthy controls, suggesting the potential involvement of CpG methylation sites in CML (Figures 1 and 2). Interestingly, two CpG sites in the intron‐1 region were found to be significantly hyper‐methylated amongst failed groups compared with newly diagnosed. In the newly diagnosed group, the frequency of CpG site methylation was significantly different from the healthy group, suggesting that CpG site methylation have a central role in the molecular events leading to CML. These findings support the assumption that the CML disease is not only driven by the *BCR‐ABL1* translocation (Lecca & Sorio, 2016). Moreover, we also observed a significantly higher methylated CpG sites in the failed group compared to the healthy group, indicating that CpG site methylation may be important for disease progression (Table 3).

Several studies have documented the effect of DNA methylation pattern of regulatory genes on various cellular activities such as cell proliferation and survival, as well as cell‐signaling molecules in CML (Behzad et al., 2018). Jelinek et al., 2011 studied the Methylation levels of 10 genes in CML patients and found that the frequency of methylated genes ranged from 11%to 86% as follows: *ABL1* (86%), *CDH13* (79%), *NPM2* (74%), *PGRA* (66%), *TFAP2E* (63%), *DPYS* (54%), *PGRB* (52%),*OSCP1* (30%), *PDLIM4* (21%), and *CDKN2B* (11%), suggesting an aberrant methylation of DNA associated with the progression of the disease (Jelinek et al., 2011). Another study using a whole methylome approach in 36 CML patients, found that 31 genes were uniquely hyper methylated in CML and 42 genes that became hyper methylated with the progression of CML. Remarkably, the same group showed that utilizing DNA methylation inhibitor such as azacytidine in blastic crisis CML patients resistant to Imatinib Mesylate (IM) could reverse the aberrant hypermethylation associated with progression of CML to blast crisis and supports the use of this drug as an epigenetic therapy (Byun, 2007). In another study with CML cell line K562 and its IM resistant variant (K562‐R) the methylation levels were found to be significantly higher and that the gene expression levels were significantly lower for *MLH1, RPRM*, *FEM1B*, and *THAP2* in K562‐R cells when compared to parental K562 cells. Further, treatment of the K562‐R cells with epigenetic drugs, such as 5‐azacytidine (AzaC) reduced resistance to Imatinib Methylate (REN, 2012). In another study, *SOX30* methylation has been correlated with disease progression in patients with chronic myeloid leukemia (Zhang et al., 2019).

Protein tyrosine phosphatase receptor gamma expression has been shown to be downregulated by RAS activation, while its upregulation has been observed in hypo‐methylation condition in in childhood acute lymphoblastic leukemia (ALL) (Xiao et al., 2014). Finally, *PTPRG* methylation has also been reported in solid cancer (Cheung et al., 2008; Wang & Dai, 2007). Eddy *et al.,* suggested that *PTPRG* inton1 methylation could be a biomarker for early detection of colorectal cancer (van Roon et al., 2011).

## 5 CONCLUSION

Hypermethylation of *PTPRG* locus might suggest a molecular mechanism independent of *BCR‐ABL1* function in CML patients. Our data contributes to deepen our understanding of the crucial role of aberrant DNA methylation in CML disease initiation and progression. Potentially, *PTPRG* methylation could be a biomarker for early detection of CML. However, further studies are needed on the validation of specific aberrant methylation of *PTPRG* and its prognostic and predictive values for the response to therapy in the CML patients.

## CONFLICTS OF INTEREST

The authors declare that there are no conflicts of/or competing interests.

## AUTHORS' CONTRIBUTIONS

MI was responsible for performing the experiments; MI, MS, and ND were responsible for analyzing the data. MI and ND designed the experiment. MI, ND, and MS performed statistical analysis. MI, MY, and ND involved in patient's recruitment. MI, ND, AS, MS, MV, GV, MM, MS, HiM, MWQ, HZ, RC, CS, HeM, and ND conceptual work, framework, draft write‐up and editing. All authors read and approved the final manuscript.

## ETHICAL APPROVAL AND CONSENT APPROVAL

Informed consent was obtained from all participants. The study was approved by both Ministry of Public Health and institutional review board of Hamad Medical Corporation (Project No. 1118/11). This study adhered to the World Medical Association's Declaration of Helsinki (1964–2008) for Ethical Human Research including confidentiality, privacy, and data management.

## CONSENT FOR PUBLICATION

Consent for publication was obtained through ethics approval and consent to participate.

## Supporting information

Tables S1‐S3

## Data Availability

This is a research article and all data generated or analyzed during this study are included in this publication All enquiries should be directed to Dr. Nader Al‐Dewik: naldewik@hamad.qa.
