# Supplementary material for: Aberrant DNA methylation of PTPRG as one possible mechanism of its under‐expression in CML patients in the State of Qatar
Source: Mol Genet Genomic Med. 2020 Jul 23;8(10):e1319. doi: 10.1002/mgg3.1319 (PMC7549574; doi:10.1002/mgg3.1319)
Supplement: Supplementary file 1 — Tables S1‐S3 [file MGG3-8-e1319-s001.docx]

| **PTPRG Promoter CpG island** | | **PCR product** |
| --- | --- | --- |
| **Forward primer** | Length**:** 22bp.  5' AGAGAGTAGAGTYGAGGGATTT 3'  Tm=58.94; CpG=1; C=5 | Length: 218 bp.  5' AGAGAGTAGAGTYGAGGGATTTAGYGTAAGGYGGGAGTTAAGYGYGGTTGTTTTAAGAAYGYGGAGAGYGYGYGTTYGTYGTTAGTTGGTTYGGGTTGYGYGTTTTYGTYGTTATYGYGYGTTTTTTGTTYGTTYGTTTTTTYGTTYGTYGGTTTTAAAGTTTTTGTTAGGATTTATGTTTATATGTTATTTTTTGTATGGAGGTATGGTTAGTTTTT 3'  %CGs=42.66  25 CpG sites |
| **Reverse primer** | Length: 24 bp.  5' AAAACTAACCATACCTCCATACAA 3'  Tm=58.96; CpG=0; C=5 |  |
| **PTPRG Intron-1 CpG island** | | **PCR product** |
| **Forward primer** | Length: 22bp.  5' GAGAGGGAGTAGTAGGTTTTGG 3'  Tm=59.54; CpG=0; C=3 | Length: 321 bp.  5' GAGAGGGAGTAGTAGGTTTTGGAGTAAGGTAAAGTTAAAATATTAGAGTTTTGGGAGAYGTTGGTTTTTYGTTTTTYGAGGTTGTYGYGAYGTYGTTGGATTTTAGGGGGYGTTTTYGAGTTATTGGTGGGGTTTTTGTTATTTTTATATTGGTYGGTTTYGGTTATTTTTAYGTTTTAGGGATGGGGYGYGYGTGTTYGGGTTTTTAGGTTTTGGGGTTGTAGAYGYGTTTTGGYGYGAGGYGGYGGTTTYGGTTTGGYGATGTTGTTTTTGGTTTTTTYGAAATTYGTTGGGGGTTGGAGTTAGTTTTGGGATTTTTAT 3'  %CGs=43.61  26 CpG sites |
| **Reverse primer** | Length: 24 bp.  5' ATAAAAATCCCAAAACTAACTCCA 3'  Tm=60.09; CpG=0; C=7 |  |

**Supplementary Table1:** Primer designing report with possible CpG sites for Promoter and interon-1

| **Gel QC for Promoter PTPRG CpG Island** | **Gel QC for Intron-1 PTPRG CpG Island** |
| --- | --- |
| 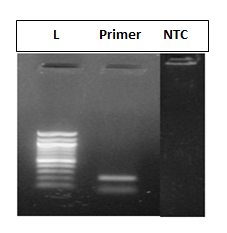 | 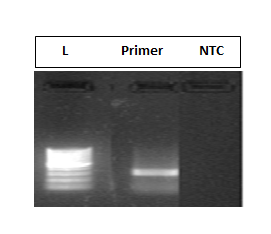 |
| 218bp specific product at 60ºC | 321bp specific product at 60ºC |

**Supplementary Table 2**: 218bp& 321Products of PCR of Promoter and Intron-1 respectively

| **CpG sites of Promoter** | **Genomic Co-ordinate** |
| --- | --- |
| CpG 13 | Chromosome 3: 61,561,400 |
| CpG 15 | Chromosome 3: 61,561,412 |
| CpG 32 | Chromosome 3: 61,561,419 |
| CpG 43 | Chromosome 3: 61,561,430 |
| CpG 45 | Chromosome 3: 61,561,432 |
| CpG 60 | Chromosome 3: 61,561,447 |
| CpG 62 | Chromosome 3: 61,561,449 |
| CpG 69 | Chromosome 3: 61,561,456 |
| CpG 71 | Chromosome 3: 61,561,458 |
| CpG 73 | Chromosome 3: 61,561,460 |
| CpG 77 | Chromosome 3: 61,561,464 |
| CpG 80 | Chromosome 3: 61,561,467 |
| CpG 92 | Chromosome 3: 61,561,479 |
| CpG 99 | Chromosome 3: 61,561,486 |
| CpG 101 | Chromosome 3: 61,561,488 |
| CpG 107 | Chromosome 3: 61,561,494 |
| CpG 110 | Chromosome 3: 61,561,497 |
| CpG 116 | Chromosome 3: 61,561,503 |
| CpG 118 | Chromosome 3: 61,561,505 |
| CpG 120 | Chromosome 3: 61,561,507 |
| CpG 131 | Chromosome 3: 61,561,518 |
| CpG 135 | Chromosome 3: 61,561,522 |
| CpG 143 | Chromosome 3: 61,561,530 |
| CpG 147 | Chromosome 3: 61,561,534 |
| CpG 150 | Chromosome 3: 61,561,537 |

| **CpG sites of Intron-1** | **Genomic Co-ordinate** |
| --- | --- |
| CpG 59 | Chromosome 3: 61,564.673 |
| CpG 70 | Chromosome 3: 61,564,684 |
| CpG 77 | Chromosome 3: 61,564,691 |
| CpG 86 | Chromosome 3: 61,564,700 |
| CpG 88 | Chromosome 3: 61,564,702 |
| CpG 91 | Chromosome 3: 61,564,705 |
| CpG 94 | Chromosome 3: 61,564,,708 |
| CpG 111 | Chromosome 3: 61,564,725 |
| CpG 117 | Chromosome 3: 61,564,731 |
| CpG 155 | Chromosome 3: 61,564,769 |
| CpG 161 | Chromosome 3: 61,564,775 |
| CpG 173 | Chromosome 3: 61,564,787 |
| CpG 189 | Chromosome 3: 61.564,803 |
| CpG 191 | Chromosome 3: 61,564,805 |
| CpG 193 | Chromosome 3: 61,564,807 |
| CpG 199 | Chromosome 3: 61,564,812 |
| CpG 226 | Chromosome 3: 61,564,840 |
| CpG 228 | Chromosome 3: 61,564,842 |
| CpG 236 | Chromosome 3: 61,564,850 |
| CpG 238 | Chromosome 3: 61,564,852 |
| CpG 243 | Chromosome 3: 61,564,857 |
| CpG 246 | Chromosome 3: 61,564,860 |
| CpG 252 | Chromosome 3: 61,564,866 |
| CpG 260 | Chromosome 3: 61,564,874 |
| CpG281 | Chromosome 3: 61,564,895 |
| CpG 288 | Chromosome 3: 61,654,902 |

**Supplementary Table 3** Genomic Co-ordinate for possible 25 CpG sites of Promoter and possible 26 CpG sites of intron-1 of PTPRG
